# Biological Response of Human Gingival Fibroblasts to Zinc-Doped Hydroxyapatite Designed for Dental Applications—An In Vitro Study

**DOI:** 10.3390/ma16114145

**Published:** 2023-06-02

**Authors:** Madalina Andreea Badea, Mihaela Balas, Marcela Popa, Teodora Borcan, Anamaria-Cristina Bunea, Daniela Predoi, Anca Dinischiotu

**Affiliations:** 1 Department of Biochemistry and Molecular Biology, Faculty of Biology, University of Bucharest, 91-95 Splaiul Independentei, 050095 Bucharest, Romania; madalina-andreea.badea@bio.unibuc.ro (M.A.B.); mihaela.balas@bio.unibuc.ro (M.B.); borcan.teodora@s.bio.unibuc.ro (T.B.); bunea.anamaria_cristina@s.bio.unibuc.ro (A.-C.B.); 2 Research Institute of the University of Bucharest (ICUB), University of Bucharest, 90-92 Sos. Panduri, 050663 Bucharest, Romania; 3 Department of Botany and Microbiology, Faculty of Biology, University of Bucharest, 1-3 Aleea Portocalelor, 060101 Bucharest, Romania; marcela.popa@bio.unibuc.ro; 4 National Institute of Materials Physics, No. 405A Atomistilor Street, 077125 Magurele, Romania; dpredoi@gmail.com

**Keywords:** hydroxyapatite, zinc, gingival fibroblasts, biocompatibility, dental applications

## Abstract

This study aimed to investigate the biological response induced by hydroxyapatite (HAp) and zinc-doped HAp (ZnHAp) in human gingival fibroblasts and to explore their antimicrobial activity. The ZnHAp (with xZn = 0.00 and 0.07) powders, synthesized by the sol-gel method, retained the crystallographic structure of pure HA without any modification. Elemental mapping confirmed the uniform dispersion of zinc ions in the HAp lattice. The size of crystallites was 18.67 ± 2 nm for ZnHAp and 21.54 ± 1 nm for HAp. The average particle size was 19.38 ± 1 nm for ZnHAp and 22.47 ± 1 nm for HAp. Antimicrobial studies indicated an inhibition of bacterial adherence to the inert substrate. In vitro biocompatibility was tested on various doses of HAp and ZnHAp after 24 and 72 h of exposure and revealed that cell viability decreased after 72 h starting with a dose of 31.25 µg/mL. However, cells retained membrane integrity and no inflammatory response was induced. High doses (such as 125 µg/mL) affected cell adhesion and the architecture of F-actin filaments, while in the presence of lower doses (such as 15.625 µg/mL), no modifications were observed. Cell proliferation was inhibited after treatment with HAp and ZnHAp, except the dose of 15.625 µg/mL ZnHAp at 72 h of exposure, when a slight increase was observed, proving an improvement in ZnHAp activity due to Zn doping.

## 1. Introduction

Hydroxyapatite (HAp) is a calcium phosphate mineral with the chemical formula Ca_10_(PO_4_)_6_(OH)_2_, which occurs naturally and belongs to the apatite general group [1]. HAp is mainly found in teeth and bones as crystals with nanometric dimensions and possesses good biocompatibility, bioactivity, and osteogenic capacity [2]. In bones, HAp presents a length of 60 nm and a width of 5–20 nm, while in mature dentine, its dimensions are a length of 60–70 nm, thickness of 3–4 nm, and width of 20–30 nm [3]. In recent years, significant attention has been paid to HAp nanoparticles (NPs), which have been explored for their roles in many biomedical areas: coating and composite material, drug delivery carriers, ceramics [4], bio-imaging agents [5], and bone substitutes [6]. Moreover, considerable attention has been paid to the use of HAp NPs in prophylactic, regenerative, aesthetic, conservative, and restorative dentistry due to the high concentration of calcium present in their composition [3]. Moreover, HAp NPs present re-mineralizing effects on enamel lesions [7,8] and bioactivity, and promote bone–implant contact, osteointegration, and osteogenesis processes at the implantology site [9,10]. In dentistry, HAp NPs are used to counteract dentin hypersensitivity or caries development, and in implantology procedures when they are used as a coating material of the titanium implant [11]. HAp NPs also find application in the reconstruction of periodontal defects by repairing the fibrous connective tissue surrounding the granules [12,13]. Additionally, HAp can be used as a direct pulp capping agent and in pulpotomy, because of its osteoconductive property, and as a dental drug delivery tool for periodontitis treatment [14].

For use in specific applications and to improve re-mineralization properties, HAp’s physiochemical properties and biological activity can be modulated by the adsorption of organic molecules on its surface or by ion substitution [15]. HAp can incorporate a variety of cations (Sr^2+^, Mg^2+^, Zn^2+^, Na^+^, K^+^, Li^+^, Ag^+^, Fe^2+^, Mn^2+^, and Cu^2+^) or anions (CO_3_^2−^, F^−^, SiO_4_^4−^, SeO_3_^2−^, and SeO_4_^2−^) [16].

Zinc is an essential trace element, with primordial roles in many enzymes’ activity, cell division, DNA and protein synthesis, immune function, and bone mineralization [17]. HAp substitution with Zn^2+^ has significant importance in the field of dental applications because of the implications of zinc in oral health. In the oral cavity, zinc is present in saliva, dental plaque, and dental hard tissues. Among the benefits of zinc in dental health, preventing oral diseases, maintaining enamel resistance, and preventing caries and tartar development are the most important advantages provided by this element [18,19]. It was proved that zinc–HAp toothpaste has a protective effect against enamel erosion [20]. Moreover, other complexes such as zinc–carbonate HAp are effective in enamel re-mineralization and reduction in dentin erosion [21].

Moreover, Zn^2+^-substituted HAp has been studied because of its effective antibacterial response, as demonstrated in various Gram-positive and Gram-negative strains: *Escherichia coli*, *Staphylococcus aureus* [22], *Bacillus subtilis*, *Enterobacter aerogenes*, etc. [23].

Due to the promising advantages provided by Zn doping, various methods have been developed for the synthesis of zinc-doped HAp (ZnHAp): the sol-gel method, co-precipitation method, hydrothermal synthesis, and solid-state methods [24], with each method leading to different morphologies, sizes, and crystallinities of HAp. Besides chemical synthesis, HAp can also be obtained by extraction from natural HAp sources. Additionally, due to the nanometric dimensions of HAp in human bones and teeth, concern about obtaining HAp NPs has increased in recent years [25]. Besides the advantages provided by ion substitution, in-depth studies are necessary to demonstrate the biocompatibility of ZnHAp in various cell types and its antibacterial properties. Thus, in this work, we focused on the synthesis of zinc-doped hydroxyapatite using the sol-gel method, physiochemical characterization, investigation of its antimicrobial activity, and in vitro biocompatibility with human gingival fibroblasts in comparison with free HAp, examining cell viability and morphology, cell proliferation and adhesion, membrane integrity, and inflammatory response.

## 2. Materials and Methods

### 2.1. Synthesis and Characterization of Hydroxyapatite and Zinc-Doped Hydroxyapatite

#### 2.1.1. Zinc-Doped Hydroxyapatite

Zinc-doped hydroxyapatite, (Ca_10−x_Zn_x_(PO_4_)_6_(OH)_2_) with xZn = 0.00 (HAp) and 0.07 (ZnHAp) were obtained using the adapted sol-gel method [26,27]. The [Ca + Zn]/P molar ratio was 1.67. The solutions of (NH_4_)_2_∙HPO_4_ and Zn(NO_3_)_2_·6H_2_O (Sigma Aldrich, St. Louis, MO, USA) were dropped into the solution of Ca(NO_3_)_2_∙4H_2_O (Sigma Aldrich, St. Louis, MO, USA). The pH was kept constant at 11 throughout the drip by adding NH_4_OH. The resulting suspension was stirred continuously for 48 h at 100 °C. The resulting gels were washed five times with double-deionized water and ethanol [28]. After washing, the gel was dispersed in deionized water under continuous stirring for 6 h. After that, the gel was dried in an oven at a temperature of 100 °C. The resulting final powder was analyzed from physicochemical and biological points of view.

#### 2.1.2. Physiochemical Characterization

To measure the crystal size of the samples, the X-ray diffraction pattern was analyzed. The pattern was obtained using a Rigaku SmartLab 3 kW (Rigaku, Tokyo, Japan) diffractometer with Kα Cu radiation (λ = 1.5418 Å), and an incidence angle of 0.5° [29]. The patterns were achieved in the 2θ range of 20–60°, and angle variation of 0.02°, with a total detector data acquisition time of 8.5 s. A scanning electron microscope (FEI Quanta Inspect F) equipped with an energy-dispersive X-ray (EDX) attachment was used to study the morphology of the analyzed samples. The particle size distribution was also obtained from the SEM micrographs. FTIR spectra of samples were collected on an SP 100 Perkin Elmer FTIR spectrometer (Waltham, MA, USA). The parameters used for the spectrum acquisition were described elsewhere [27]. In the analysis of the size distribution, the Chi-Sqr test was used. Standard error was scaled with the square root of reduced Chi-Sqr.

### 2.2. In Vitro Biocompatibility Assessment

**Figure 1:**
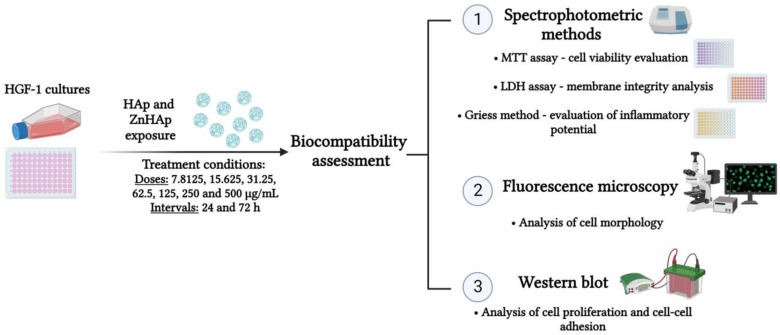
Schematic representation of the experimental design used for biocompatibility assessment. Diagram created in BioRender.com.

Figure 1 illustrates the experimental design used for the biocompatibility evaluation of HAp and ZnHAp NPs.

#### 2.2.1. Cell Culture Conditions

Human gingival fibroblasts HGF-1 (CRL-2014, ATCC, Manassas, VA, USA) are normal adherent cells and were isolated from the gingiva of a 28-year-old male. Cells were cultured in a humidified incubator at 37 °C, 95% air, and 5% CO_2_, using Dulbecco’s Modified Eagle’s Medium (DMEM, 31600-083) supplemented with 1.5 g/L NaHCO_3_, 3.5 g/L glucose, 1% antibiotic-antimycotic mix (10,000 units penicillin, 10 mg streptomycin, and 25 μg amphotericin B per mL; A5955, Sigma-Aldrich), and 10% heat-inactivated fetal bovine serum (10270-106, origin of South America, Gibco, Life Technologies, Carlsbad, CA, USA). Passage cell culture was performed using a 0.25% trypsin–0.53 mM EDTA solution and the culture medium was completely replaced every two days. For the determination of the total cell number from cultures, a hemocytometer was used. 

#### 2.2.2. Treatment Conditions

HGF-1 cells were seeded in culture plates and flasks, incubated for 24 h for cell adherence, and then exposed to various doses of HAp and ZnHAp NPs for 24 and 72 h. The following doses of HAp and ZnHAp were tested: 7.8125, 15.625, 31.25, 62.5, 125, 250, and 500 µg/mL. Untreated cells were used as control. Before treatment, NP suspensions were sterilized by exposure to a UVC lamp for 1 h. 

#### 2.2.3. Cell Viability Evaluation

Cell viability of HGF-1 cells was evaluated using the MTT assay based on the reduction by mitochondrial dehydrogenases of the tetrazolium dye MTT to a violet formazan. The cells were seeded in 96-well plates (3.25 × 10^4^ cells/mL), incubated overnight, and then treated with HAp and ZnHAp suspensions. After 24 and 72 h of incubation, the medium was completely aspired, the cell monolayer was washed with phosphate buffer saline (PBS), and incubated with a 1 mg/mL MTT solution for 2 h, 37 °C, in the dark. Afterwards, the MTT solution was discarded and the formazan crystals were dissolved in 120 µL isopropanol. Coloration intensity was estimated by absorbance spectrophotometry at a wavelength of 595 nm, using a Tecan GENios microplate reader (Tecan Trading AG, Männedorf, Switzerland). The results were calculated as percentages (%) of control (100%, untreated cells). 

#### 2.2.4. Lactate Dehydrogenase (LDH) Activity Assessment

The level of LDH activity released in the culture medium was measured after the treatment of HGF-1 cells with 15.625 and 125 µg/mL HAp and ZnHAp for 24 and 72 h, according to the instructions of the manufacturer of the commercial colorimetric assay kit, “Cytotoxicity Detection Kit”, purchased from Roche (cat. no. 11644793001, Basel, Switzerland). In brief, 50 µL culture medium from treated and control cells was homogenized with 50 µL reaction mixture (catalyst and dye solution 1:45) and then incubated for 20 min at room temperature, in dark conditions. In the end, the absorbance was measured at Tecan GENios, 485 nm wavelength.

#### 2.2.5. Measurement of Nitric Oxide (NO) Production

The production of nitric oxide (NO) in the culture medium of cells exposed to 15.625 and 125 µg/mL HAp and ZnHAp was measured using the Griess method. After 24 and 72 h of treatment, 80 µL of culture medium was treated with 80 µL Griess Reagent (0.1% naphthyl-ethylene-diamine:1% sulfanilamide 1:1) and then the absorbance was read at 550 nm, using a Tecan GENios microplate reader. Finally, the absorbance values were extrapolated on a standard curve (0–100 µM NaNO_2_) and calculated related to the control (untreated cells). 

#### 2.2.6. Analysis of F-Actin Cytoskeleton Organization

Organization of the F-actin cytoskeleton after 24 and 72 h of treatment with 15.625 and 125 µg/mL HAp and ZnHAp was analyzed by fluorescence microscopy, using an Olympus IX73 microscope (Olympus, Tokyo, Japan). For the labeling of F-actin cytoskeleton and nuclei, HGF-1 cells were seeded in 24-well plates (3.25 × 10^4^ cells/mL), incubated overnight for cell adherence, and then treated with 15.625 and 125 µg/mL HAp and ZnHAp NPs. At the chosen intervals, the medium was removed, the cells were washed with PBS and fixed with 4% paraformaldehyde (20 min, 4 °C). Further, the paraformaldehyde solution was removed and cells were washed with PBS. The cell membrane was permeabilized with 0.1% Triton X-100 and 2% bovine serum albumin (BSA) solution prepared in PBS (45 min, room temperature, shaking). Subsequently, cells were incubated with 150 nM Alexa FluorTM 488 phalloidin (prepared in PBS containing 1.2% BSA; A12379, Invitrogen by Thermo Fisher Scientific, Eugene, OR) for F-actin filament staining (45 min, room temperature). Then, cells were washed with PBS and the nuclei were labeled using a 2 µg/mL Hoechest 33342 solution (H3570, Life Technologies, Carlsbad, CA, USA) prepared in PBS (10 min, room temperature). Finally, cells were washed with PBS and then analyzed under a fluorescence microscope using green and blue filters. 

#### 2.2.7. Western Blot

HGF-1 cells were cultured in 75 cm^2^ treated culture flasks (5 × 10^5^ cells/flask) and exposed to 15.625 and 125 µg/mL HAp and ZnHAp suspensions. At 24 and 72 h of incubation, cells were enzymatically detached from the culture surface, washed, and resuspended in PBS. Then, they were lysed on ice through ultrasonication (three times, 30 s) using a UP50H ultrasonicator (80% amplitude, 1 cycle, Hielscher Ultrasound Technology, Teltow, Germany) and the resulted homogenates were centrifuged for 10 min, at 3000 rpm, 4 °C. Finally, supernatants were aliquoted and used for the Western blot technique. The protein content of cell lysates was measured using Bradford assay, using a 3 mg/mL bovine serum albumin solution as standard (0–1.5 mg/mL standard curve).

After chemical and thermic denaturation (5 min, 95 °C) of proteins, equal amounts (40 μg) were loaded on 8% sodium dodecyl sulfate (SDS)-polyacrylamide gels and subjected to electrophoresis in running buffer (0.05 M Tris, 0.05 M glycine, and 0.1% SDS) at 90 V. Then, the proteins were electroblotted (350 mA) onto polyvinylidene difluoride (PVDF) membranes (cat. no. IPVH00010, Merck, Darmstadt, Germany) using a transfer buffer containing 25 mM Tris, 192 mM glycine, and 20% (*v*/*v*) methanol. Both electrophoresis and electroblotting were performed using Bio-Rad systems. 

Further, a Western Breeze Chromogenic Anti-Mouse kit (WB7103, Invitrogen, Carlsbad, CA, USA) was used for protein band detection. Membranes were blocked for 30 min, at room temperature, and then incubated overnight with anti-MCM-2 (sc-373702, Santa Cruz, Dallas, TX, USA) and anti-β-catenin (sc-59737, Santa Cruz, Dallas, TX, USA) primary monoclonal antibody solutions (1:250 dilution). To confirm equal protein loading, each membrane was simultaneously incubated with an anti-β-actin primary monoclonal antibody (A1978, Sigma-Aldrich, St. Louis, MO, USA). The next day, membranes were washed three times with wash solution (5 min), incubated with conjugated-alkaline phosphatase anti-mouse second antibody solution (30 min, room temperature), and then washed two times. The signals were detected using BCIP/NBT chromogenic substrate, visualized with a ChemiDoc Imaging System (Bio-Rad, Hercules, CA, USA), and quantified using ImageLab software (version 6.1.0, Bio-Rad, Hercules, CA, USA). The results were normalized to the reference protein (β-actin). 

#### 2.2.8. Statistical Analysis

All determinations were performed in triplicate. Data were analyzed using GraphPad Prism 8.0.2 software (San Diego, CA, USA) and statistical differences between control and treated groups were calculated using one-way analyses of variance (ANOVA) for the MTT test and two-way ANOVA for LDH, NO, and Western blot determinations, followed by Tukey’s multiple comparison test. Results were considered statistically significant when *p* < 0.05.

### 2.3. Antimicrobial Activity Evaluation

The antimicrobial assays were performed on Gram-positive (*Staphylococcus aureus* ATCC 25923, *Enterococcus faecalis* ATCC 29212) and Gram-negative (*Escherichia coli* ATCC 25922, *Pseudomonas aeruginosa* ATCC 27853) bacterial strains (American Type Culture Collection [ATCC]). 

The quantitative assay of the antimicrobial activity was performed using the liquid medium microdilution method, in 96-multi-well plates, to establish the minimal inhibitory concentration (MIC). Hence, serial twofold dilutions of the compounds ranging between 0.145–74 mg/mL HAp and 0.03–15.39 mg/mL ZnHAp were performed in Tryptone Soy Broth, and each well was seeded with 10^6^ CFU/mL (Colony Forming Unit, CFU). Sterility control (wells containing only culture medium) and culture controls (wells containing culture medium seeded with the microbial inoculum) were used. The plates were incubated for 24 h at 37 °C, and MIC values were considered as the lowest concentration of the tested compound that inhibited the visible growth of the microbial overnight cultures.

The assessment of the compounds’ influence on the microbial ability to colonize an inert substratum to form biofilms was performed by the microtiter method; briefly, after MIC determination, the plates were discarded, washed, stained with crystal violet, and treated with acetic acid. The absorbance at 490 nm was measured with a Thermo Scientific Multiskan FC Microplate Photometer.

Experiments were performed in duplicate.

## 3. Results

### 3.1. Characterization of Hydroxyapatite and Zinc-Doped Hydroxyapatite

**Figure 2:**
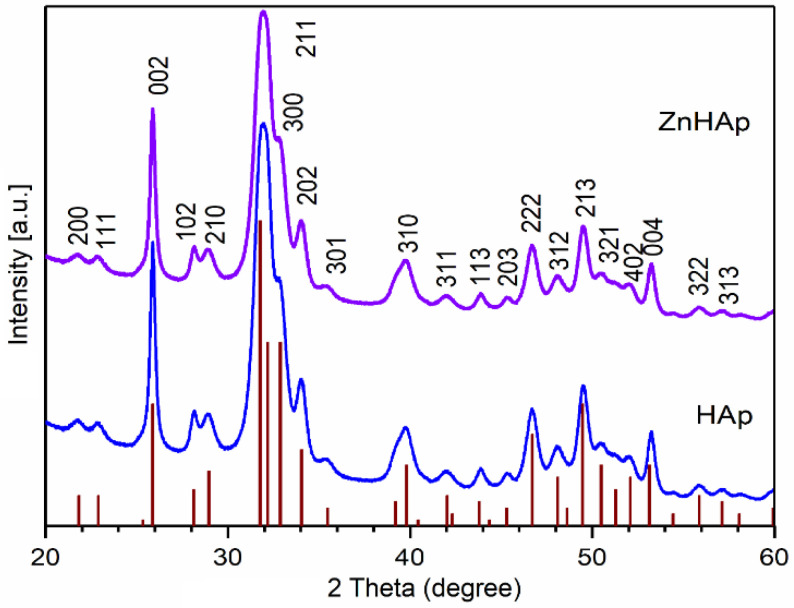
XRD pattern of HAp (xZn = 0.00) and ZnHAp (xZn = 0.07) powders. Standard pattern of the pure hexagonal HAp (JCPDF 9-432; vertical lines).

The XRD patterns of prepared HAp and ZnHAp powders, and that of pure HAp (vertical lines), are presented in Figure 2. The diffraction peaks of HAp and ZnHAp powders correspond to the vertical lines that represent the positions of diffraction lines of pure HAp with the reference hexagonal structure ICDD-PDF No. 9-432 [30]. Following the analysis of the diffraction spectra of the two samples, a single phase associated with pure HAp was identified. Moreover, the two samples revealed good crystallinity. The presence of zinc ions led to a slight shift in the diffraction peaks in the case of the ZnHAp sample. The crystallite sizes of the HAp and ZnHAp samples were calculated using Scherrer’s formula [31]: L = Kλ/β·cosθ, where L is nanocrystallite size, K is dimensionless shape factor, and λ (nm) is X-ray wavelength from measuring the full width at half maximum of peaks (β) in radians located at any 2θ in the pattern [32]. The size of the crystallites was smaller in the case of the ZnHAp sample (18.67 ± 2 nm) than that of the HAp sample (21.54 ± 1 nm). This fact was also observed in the width of the diffraction maxima. When the zinc concentration increased from xZn = 0.00 to xZn = 0.07, the diffraction peaks became slightly broad. Another additional phase was not identified in the analyzed spectra. The results were in accordance with the preceding studies [33,34].

**Figure 3:**
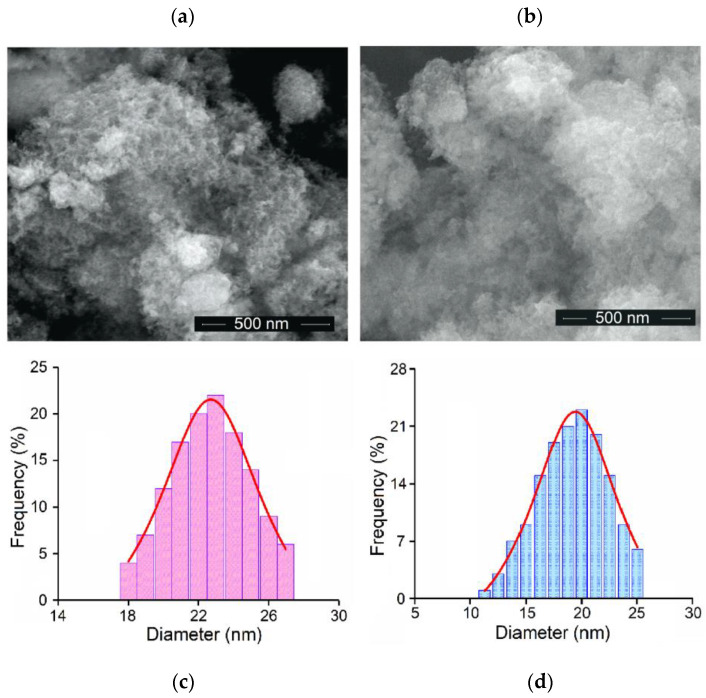
SEM micrographs of the HAp (**a**) and ZnHAp (**b**) samples at 100,000× magnification. Size distributions of the HAp (**c**) and ZnHAp (**d**) samples.

Figure 3 shows the SEM micrographs of HAp and ZnHAp samples. The HAp sample shows an acicular morphology (Figure 3a) while the ZnHAp sample presents an ellipsoidal morphology (Figure 3b). From the size distribution obtained by measuring 200 particles from the SEM micrographs, the average particle size for the two samples was calculated (Figure 3c,d). Thus, the average particle size was 22.47 ± 1 nm for HAp and 19.38 ± 1 nm for ZnHAp. The smaller size in the case of the sample doped with Zn could be due to the presence of zinc ions. Reduced Chi-Sqr was 0.29 for HAp while R-Square was 0.992. For ZnHAp, the reduced Chi-Sqr was 0.27 while the R-Square was 0.095. The results obtained by SEM are in agreement with those obtained by XRD analysis.

**Figure 4:**
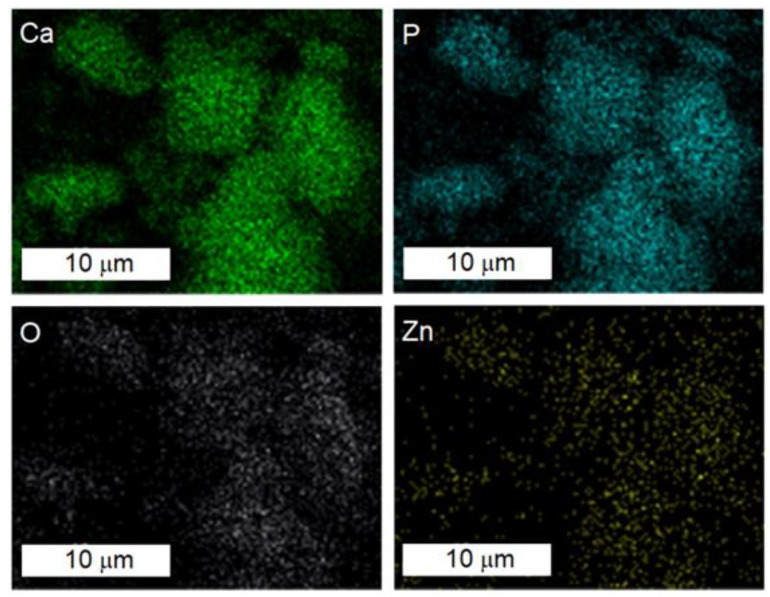
Elemental mapping of the ZnHAp (xZn = 0.07) sample. Homogeneous distribution of calcium (Ca), phosphorus (P), oxygen (O), and zinc (Zn).

The results of the chemical mapping of ZnHAp obtained using SEM analysis are presented in Figure 4. The results of elemental mapping highlighted the homogeneous distribution of the main constituents such as calcium (Ca), phosphorus (P), oxygen (O), and zinc (Zn) in the ZnHAp sample (Figure 4).

### 3.2. Biological Studies

#### 3.2.1. Biocompatibility Assessment

##### Cell Viability

The biological response induced by HAp and ZnHAp in HGF-1 cells was analyzed by evaluating cell viability, cell membrane integrity, inflammatory response, and cell morphology. Moreover, for a detailed assessment, cell proliferation and intercellular adhesion were explored. 

**Figure 5:**
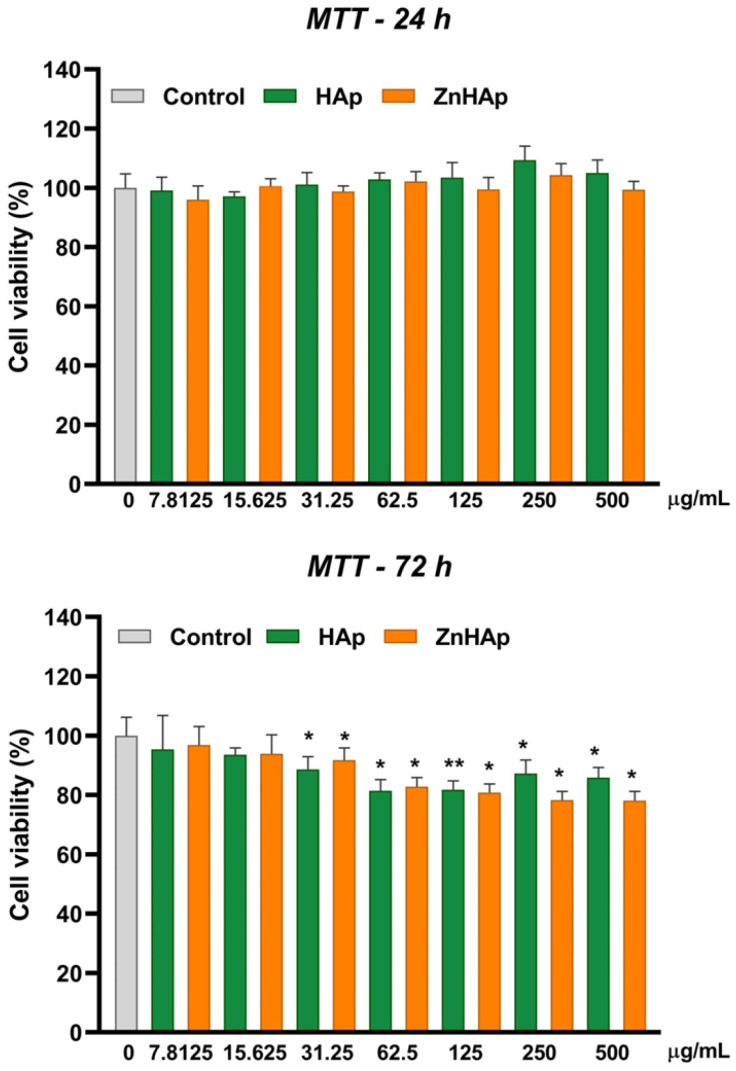
Cell viability of HGF-1 cells exposed to HAp and ZnHAp (7.8125–500 µg/mL) for 24 and 72 h. Data (*n* = 3) were calculated as mean ± standard deviation (SD) and are expressed as percentages of control (100% viability). Statistical differences between control and treated groups were calculated using one-way analyses of variance (ANOVA) and the results were considered significant when *p* < 0.05 (*); *p* < 0.01 (**) (sample vs. control).

Cell viability of cells exposed to ZnHAp was evaluated in comparison to that registered in the presence of HAp and performed by MTT assay. The cell viability was not significantly modified compared to the control after 24 h of incubation with both suspensions, which can indicate good cytocompatibility in these experimental conditions. However, at 72 h exposure, a slight decrease in cell viability starting with a dose of 31.25 µg/mL of both samples was noticed. The decrease in cell viability was dose-dependent for ZnHAp NPs up to 500 µg/mL and for HAp NPs up to 62.5 µg/mL. Higher doses of HAp induced a reduced inhibition of cell viability. The lowest level of cell viability was registered after cells’ exposure to 500 µg/mL ZnHAp (decrease by 21.87% compared to control) (Figure 5). However, no differences in cell viability between HAp and ZnHAp samples exposed to the same dose were observed. For further experiments, two doses of both samples were chosen: 15.625 µg/mL (low dose) and 125 µg/mL (high dose).

##### Cell Membrane Integrity and Inflammatory Response

**Figure 6:**
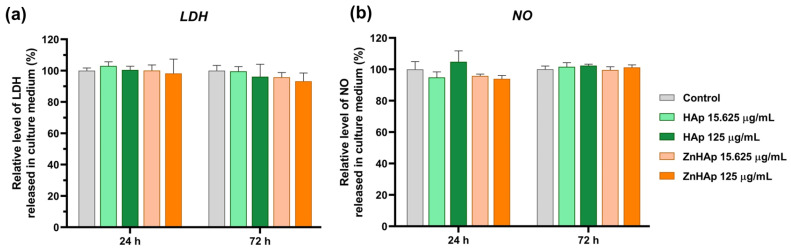
Relative levels of lactate dehydrogenase (LDH) activity (**a**) and nitric oxide (NO) (**b**) released in the culture medium of HGF-1 cells after the treatment with 15.625 and 125 μg/mL of HAp and ZnHAp, respectively, for 24 and 72 h. Data (*n* = 3) are expressed as percentages related to control ± standard deviation (SD). Statistical differences between control and treated groups were calculated using two-way analyses of variance (ANOVA).

The membrane integrity of exposed cells was assessed by measuring the level of lactate dehydrogenase (LDH) activity released in the culture medium. In normal conditions, the LDH enzyme is intracellularly located, but when the cell membrane is injured, it is released in the extracellular medium. After 24 and 72 h of exposure of HGF-1 cells to HAp and ZnHAp NPs, no increase in LDH level in the culture medium compared to control was registered (Figure 6a). For the investigation of inflammatory potential, the production of nitric oxide (NO) released in the culture medium was measured. NO is a molecule associated with inflammation when it is overproduced and released in the extracellular medium. Incubation of HGF-1 cells with 15.625 and 125 µg/mL doses for 24 and 72 h induced no significant rise in the NO level in comparison with untreated cells (Figure 6b). Both findings indicated that cell integrity was maintained and no inflammatory response was triggered in the presence of HAp and ZnHAp.

##### Cell Morphological Alterations and Protein Expression of Cell Proliferation and Adhesion-Related Markers

**Figure 7:**
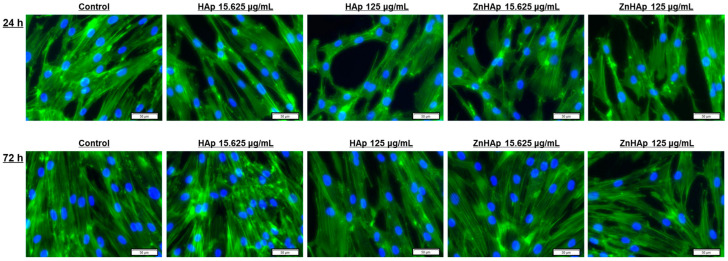
Fluorescence microscopy images with HGF-1 cells exposed to 15.625 and 125 μg/mL of HAp and ZnHAp NPs, respectively, for 24 and 72 h. Green fluorescence corresponds to the F-actin cytoskeleton and blue fluorescence indicates cell nuclei. Scale bar: 50 μm.

Cell morphology after exposure to HAp and ZnHAp NPs was analyzed by fluorescence labeling of the F-actin network and nuclei. Fluorescence staining revealed a normal and organized shape of F-actin filaments, elongated morphology of cells, and prominent nuclei specific to fibroblasts, for all experimental conditions, except the high doses of HAp and ZnHAp, when a low reduction in cell density and a slight change in the architecture of the F-actin cytoskeleton were noticed (Figure 7).

**Figure 8:**
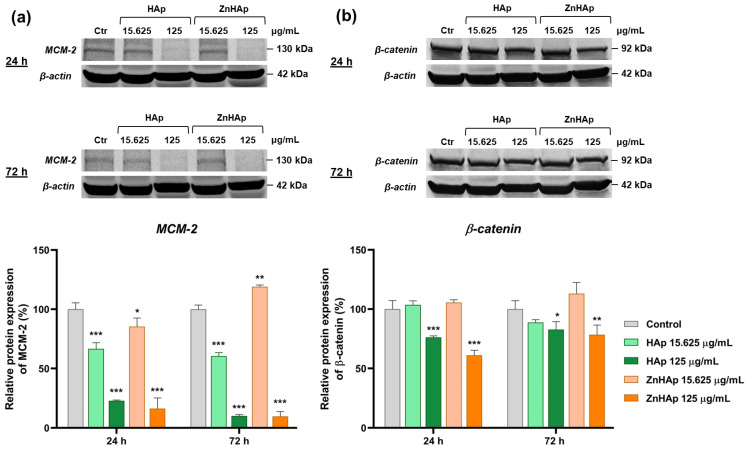
Relative protein expression of MCM-2 (**a**) and β-catenin (**b**) after the treatment with 15.625 and 125 μg/mL HAp and ZnHAp, respectively, for 24 and 72 h. Graphs represent the quantification of the corresponding bands presented above. Data (*n* = 3) are expressed as percentages related to control ± standard deviation (SD). Statistical differences between control and treated groups were calculated using two-way analyses of variance (ANOVA) and the results were considered statistically significant when *p* < 0.05 (*); *p* < 0.01 (**); *p* < 0.001 (***) (sample vs. control).

Cell proliferation was evaluated by analyzing the expression of MCM-2 protein by Western blotting (Figure 8a). After 24 h of treatment with both samples, MCM-2 protein expression decreased in a dose-dependent manner compared to untreated cells. After treatment with the low dose, the expression of MCM-2 protein was more inhibited in the presence of HAp (down-regulation by 33.4%), compared to ZnHAp (decrease by 14.6%). However, a switch in protein expression was observed between the two samples after the incubation with the high dose. After 72 h, MCM-2 protein expression was down-regulated after the incubation with doses of both HAp and 125 μg/mL of ZnHAp, compared to the control. A slight up-regulation (by 19% compared to the control) of this protein expression was registered in the presence of 15.625 μg/mL ZnHAp, showing good biocompatibility of ZnHAp in low doses.

Cell–cell adhesion in the presence of HAp and ZnHAp NPs was studied based on the expression of β-catenin protein. The dose of 15.625 μg/mL of HAp and ZnHAp induced no significant modifications of β-catenin expression compared to control, while in the presence of 125 μg/mL, the expression of this protein was down-regulated in comparison to control after 24 and 72 h. After 72 h of exposure, the expression of β-catenin protein presented a higher expression in the presence of HAp and ZnHAp compared to that registered after 24 h (Figure 8b).

#### 3.2.2. Antimicrobial Evaluation

Analyzing the antimicrobial activity of the HAp and ZnHAp NPs, we observed that these inhibit bacterial adherence to the inert substrate (Table 1), with *S. aureus* being the most susceptible to their action. Regarding the quantitative evaluation of the antimicrobial activity, the concentrations reached during testing (maximum ½ initial concentration) are too low to inhibit bacterial growth. 

## 4. Discussion

The primary stage in exploring the biocompatibility of materials designed for biomedical applications is in vitro testing. In particular, significant attention is granted to the biocompatibility of hydroxyapatite (HAp), which possesses important properties that recommend it for specific applications in dentistry [35]. In this research, we aimed to characterize zinc-doped hydroxyapatite (ZnHAp) and to study its in vitro biocompatibility in human gingival fibroblasts (HGF-1 cell line) in comparison to that of hydroxyapatite (HAp). We were focused on exploring if the doping of HAp with Zn ions improves the biological response of HGF-1 cells to HAp for its use in regenerative dentistry, implants, and cosmetic applications. Zn doping could improve HAp properties, as Zn is essential for the maintenance of optimal oral health [19] and is involved in various biological pathways, including nucleic acid metabolism, cellular protein synthesis, signal transduction, and apoptosis regulation [36]. Moreover, it was proved that HAp modified with Zn ions supports the adhesion, spreading, and proliferation of MC3T3-E1 osteoblast precursor cells [37] and has antimicrobial activity [38].

HAp continues to attract attention in the development and design of better materials for specific medical applications as a result of its ability to exchange ions in the structure. Zn-doped HAp could be used in implants to stimulate bioactivity and to ensure a better fixation in bone restoration processes, particularly because it is desired to create customized implants that increase implant–tissue integration. However, for the implants to be successful, the antimicrobial properties of HAp-based materials are essential. Recent studies [39,40] have shown that HAp doping with Zn ions could lead to the development of a biocompatible material with a good ability to integrate soft tissues, having a good antibacterial capacity. On the other hand, Zn is an essential element that stimulates the healing of soft tissue injuries [41]. Here, we showed that after optimizing the synthesis of HAp doped with Zn, a material with a structure similar to HAp was obtained, but with wider diffraction maxima. 

In this study, for the investigation of HAp and ZnHAp biocompatibility, the cell line of human gingival fibroblasts (HGF-1 cells) was chosen because these cells can be differentiated in odontoblasts and osteoblasts and produce collagen fibers in the healing process [42]. 

Cell viability assessment of HGF-1 cells revealed that both HAp and ZnHAp induced no cytotoxic effects after 24 h, but dropped cell viability after 72 h of treatment at doses higher than 31.25 µg/mL. These results indicated good cytocompatibility of HAp and ZnHAp at low concentrations and short exposure time, slight cytotoxicity at high concentrations combined with a longer exposure interval, and no significant differences between the two samples. These results do not agree with the general concept that HAp has good biocompatibility and affinity for cells and Zn doping improves HAp properties. However, similarly to our study, in other papers, negligible cytotoxicity of HAp at high concentrations was reported. Thus, the viability of mouse pre-osteoblast cells was above 80% after the treatment with concentrations lower than 128 μg/mL HAp for 1 day and decreased (40.4%) depending on the increase in HAp concentration (1024 μg/mL). This effect was associated with the agglomeration of HAp nanostructures into larger particle sizes, which induced low crystallinity [43]. In addition, Grenho and collaborators (2015) showed that after 3 days of treatment with nanostructured HAp (nanoHAp) incorporated with zinc oxide (ZnO) NPs, an inhibitory effect on the metabolic activity of MG63 cells (human osteoblast-like cells), compared to control, was registered. The presence of early and late apoptosis in cells was highlighted in the same condition [44]. HAp NPs also induced cytotoxicity and apoptosis in HepG2 cells in a size-dependent manner [45]. Cytotoxicity of nanosized HAp and its dependence on the shape and cell type was also demonstrated in other studies [46]. Instead, exposure to rod shape HAp NPs (10–300 µg/mL) did not significantly affect the cell viability of BEAS-2B, RAW 264.7, and HepG2 cell lines after 24 h exposure [47]. 

Biocompatibility assessment of HAp and ZnHAp was also based on the evaluation of lactate dehydrogenase (LDH) activity in the culture medium. LDH is a stable cytoplasmic enzyme that is rapidly released in the culture medium when the membrane is damaged. The release of LDH by damaged cells is correlated with various forms of cell death, including apoptosis and necrosis [48]. In our study, exposure of HGF-1 cells to HAp and ZnHAp induced no increase in LDH activity, which can indicate a lack of damage to the plasma membrane. Our results are supported by previous studies. Albrecht and collaborators (2009) investigated the cytotoxicity of HAp materials with different sizes and morphologies on NR8383 cells and primary alveolar macrophages. Exposure of NR8383 cells to HAp materials induced no increase in the LDH level for HAp–protein-composite and pure HAp (nano/needle-shaped, nano/rod-like, nano/plate-like morphologies) at concentrations up to 3000 µg/mL, but fine/dull needle-shaped HAp caused an increase at a dose of 3000 µg/mL. Instead, the same HAp samples and concentrations induced no cytotoxicity in primary alveolar macrophages [49]. These findings reflect the dependence of HAp cytotoxicity on sample characteristics and cell line. 

In testing the biocompatibility of HAp materials designed for dental applications, great attention is granted to the inflammatory response that could occur at the implantation site. In our study, inflammation was investigated by measuring the level of nitric oxide (NO). Exposure of HGF-1 cells to HAp and ZnHAp induced no modification of NO level in the culture medium, suggesting no activation of inflammatory response, a finding that was also highlighted in similar studies. The addition of Zn ions to HAp decreased the inflammatory process on polymorphonuclear-cells-associated acute inflammation by diminishing the levels of pro-inflammatory mediator interleukin-8 (IL-8) [50]. Furthermore, zinc-substituted HAp increased the chemotaxis and diminished the inflammatory reactions in human monocytes [51]. However, the benefits of zinc doping are dependent on its concentration, and it is noted that this element can induce an anti-inflammatory reaction in high concentrations and the reverse effect in low concentrations [50]. 

Cell morphology analysis revealed a slight change in F-actin filaments and a reduction in cell density in the presence of high doses. Cell proliferation was assessed by analyzing the expression of MCM-2 protein. MCM-2, a marker of cell proliferation, is a member of the MCM protein family, present in all phases of the cell cycle, with important roles in DNA replication and cell division [52]. After exposure of HGF-1 cells to both doses and samples for 24 h of treatment, down-regulation of MCM-2 expression was observed, indicating a slight cytotoxic character and explaining the reduction in cell density and number. However, after incubation with a dose of 15.625 μg/mL ZnHAp for 72 h, the up-regulation of MCM-2 expression was registered, while at the same interval and concentration of HAp, the expression of the protein was suppressed, which highlights the ability of cells to adapt to low concentrations of ZnHAp and reflects an advantage of HAp doping with Zn ions and its bioactivity. Similar results were obtained by Thian and collaborators (2013), who observed a higher increase in growth activity of human adipose-derived mesenchymal stem cells cultured on ZnHAp, compared to HAp [53]. The influence of dose in ZnHAp biocompatibility was also proved in MC3T3-E1 cells when a dose of 0.1 mg/mL ZnHAp sustained higher cell proliferation than the same dose of HAp, while a dose of 1 mg/mL induced cytotoxicity by decreasing cell growth rate [54]. 

Besides the significantly acute inhibition of cell proliferation and reduction in cell density, cell viability evaluated by the MTT assay registered only a slight decrease. To clarify this discordance, we examined by optical microscopy the cells treated with HAp and ZnHAp samples and incubated with MTT solution, and we observed a more pronounced color of formazan crystals formed in cells exposed to treatment compared to the control. This result could indicate stimulation of mitochondrial activity in the presence of HAp and it can be explained by the high concentration of calcium in HAp composition, which can be internalized in mitochondria. Thus, the results of the MTT assay could indicate the collective effect of decreasing cell viability and stimulation of mitochondrial activity, as a consequence of HAp cell internalization. Our hypothesis is supported by previous studies, which have shown that calcium accumulation in mitochondria leads to a cellular hyperactive metabolic state, and enhanced cytoplasmic Ca^2+^ increased metabolic viability by triggering the accumulation of Ca^2+^ in mitochondria [55].

Investigation of β-catenin, a component of cell–cell adhesion junctions, revealed inhibition of this marker expression after the treatment with a dose of 125 µg/mL HAp and ZnHAp, while after the incubation with a dose of 15.625 µg/mL, protein expression remained unchanged. β-catenin is an integral structural component of cadherin-based adherens junctions [56]. This protein controls E-cadherin-mediated cell adhesion at the plasma membrane and mediates the connection of adherens junction molecules with the actin cytoskeleton [57]. The decreased expression of this protein could indicate an inhibition of cell adhesion capacity and can be explained by the Zn concentration incorporated in HAp NPs. Previously, it was demonstrated that concentrations of 10–50 µM Zn reduced cell adhesion to fibronectin. Furthermore, Zn (5–50 μM) caused the disappearance of F-actin and loss of specific-cell morphology [58]. Consequently, inhibition of cell adhesion in the presence of ZnHAp can be correlated with the morphological changes of the F-actin network. Cell adhesion can be mediated by various factors; in previous studies it was established that this parameter is dependent on HAp morphology [59]. Moreover, cell adhesion, proliferation, and detachment strength were found to be sensitive to HAp surface roughness [60], and the reduction in cell proliferation/adhesion by HAp was associated with the contact surface [61]. In contradiction with our results, Mehta and collaborators (2005) demonstrated that HAp sustained the adhesion and spreading of keratocytes better than polytetrafluoroethylene and polyhydroxyethyl methacrylate [62]. Moreover, Zn-modified HAp coatings exhibited better attachment and spread of HGF-1 cells than did the Ag-modified coatings [63]. 

Antimicrobial evaluation studies of HAp and ZnHAp revealed inhibition of bacterial adherence to the inert substrate, with the most promising results found for *Staphylococcus aures*. Previously, the excellent antibacterial efficacy of Zn-doped HAp was proved against other species: *Staphylococcus epidermidis*, *Escherichia coli* [64], *Aggregatibacter actinomycetemcomitans*, *Fusobacterium nucleatum*, and *Streptococcus mutans* [65]. The antibacterial action of HAp is associated with the increased generation of free radicals (hydroxyl, peroxyl, oxygen anion, carbon dioxide anion), which leads to oxidative stress and causes bacterial cell death [66].

It is known that Zn-substituted HAp biocompatibility and antibacterial activity are related to Zn ion content, with the literature noting that the most promising result regarding the biocompatibility and antibacterial action are obtained in a range of 1–2% Zn^2+^ content [38], which can explain the cytotoxicity achieved in our study. 

Taken collectively, our results indicate that the biocompatibility of HAp and ZnHAp is dose-dependent. Low doses (up to 15.625 µg/mL) induced no alterations in cell morphology and viability, disruption of membrane integrity, modifications in cell adhesion, or activation of inflammation, but inhibited cell proliferation. High doses (125 µg/mL) decreased cell proliferation rate, inhibited cell–cell adhesion marker expression, and affected F-actin cytoskeleton filaments, but induced no inflammatory response or membrane leakage. However, a slight advantage provided by Zn doping was indicated by the up-regulation of the cell proliferation marker at low doses. Besides this, new studies regarding the adjustment of HAp and ZnHAp biocompatibility and Zn-incorporated concentration, for their applications in the dental field, are needed, as the overall study indicated a lack of biocompatibility.

An important aspect regarding the testing of HAp biocompatibility is its association in dental materials with main components, which can also influence the biological response. Frequently, HAp is embedded in biopolymer matrices (collagen, gelatin, alginate, and chitosan) or synthetic polymers (poly-ε-caprolactone, poly(3-hydroxybutyrate, poly(lactic-co-glycolic) acid) to improve biocompatibility and develop HAp-based biocomposites [67,68]. Furthermore, cross-linking agents (e.g., transglutaminases, glutaraldehyde, 1,4-butanediol diglycidyl ether, genipin, and 1-ethyl-3-(3-dimethylamino propyl) carbodiimide hydrochloride) can be used to generate a composite with an independent function [68,69]. All these components of the composites induce separate biological responses and their association could have different impacts on cells. Therefore, the evaluation of HAp cytotoxicity without the major components of composites could not lead to a comprehensive assessment. However, this study could contribute to the understanding of HAp cytotoxicity and biocompatibility for improving the synthesis methods. Furthermore, advanced tridimensional cell culture models could be more adequate for in-depth studies on signaling pathways involved in cell proliferation and adhesion in the presence of HAp and ZnHAp NPs, which would lead to a closer perspective of in vivo exposure.

## 5. Conclusions

In the present work, a facile sol-gel synthesis technique was used to obtain ZnHAp samples (xZn = 0.00 and xZn = 0.07). This technique enabled the development of ZnHAp nanocomposites having the hexagonal structure of pure HAp. The HAp (xZn = 0.00) sample showed acicular morphology. When xZn = 0.07 (ZnHAp), the morphology of the sample was ellipsoidal. The uniform dispersion of Zn ions within the HAp lattice was confirmed by elemental mapping using EDS analysis. 

In vitro biocompatibility assessment led to the following main conclusions:

- Cell viability remained at the control level after 24 h of treatment with 7.8125–500 µg/mL HAp and ZnHAp; after 72 h, a slight decrease in cell viability was registered starting with a dose of 31.25 µg/mL HAp and ZnHAp;
- Cells maintained their membrane integrity and no inflammatory response was triggered in the presence of HAp and ZnHAp;
- A dose of 15.625 µg/mL HAp and ZnHAp presented good biocompatibility, as indicated by the lack of alteration of F-actin cytoskeleton organization and unchanged protein expression of β-catenin cell adhesion marker;
- A dose of 125 µg/mL promoted the decrease in β-catenin protein expression and changed the architecture of F-actin filaments after 24 and 72 h;
- Cell rate proliferation was decreased in all experimental conditions, except for the dose of 15.625 µg/mL ZnHAp at 72 h, when a slight increase was induced, proving an improvement in HAp properties provided by zinc substitution.

The antimicrobial evaluation indicated an inhibition of the bacterial adherence to the inert substrate. 

Considering our results, further studies are needed to increase HAp and ZnHAp biocompatibility at high doses to improve their use in dental applications. 

## Figures and Tables

**Table 1:** Values of the minimum concentrations that inhibit bacterial adherence to the inert substrate.

Compound	*S. aureus*		*E. faecalis*		*E. coli*		*P. aeruginosa*	
HAp (mg/mL)	4.625	4.625	No	No	37	9.25	9.25	9.25
ZnHAp (mg/mL)	0.962	0.481	3.848	3.848	No	No	3.848	3.848

## Data Availability

Not applicable.
